# The decision-making process for sedation in specialist palliative care: a qualitative interview study with team members, relatives, and patients

**DOI:** 10.1186/s12904-026-02029-9

**Published:** 2026-02-24

**Authors:** Violet Handtke, Sophie Meesters, Jeremias Bazata, Jan Schildmann, Claudia Bozzaro, Christoph Ostgathe, Claudia Bausewein, Carsten Klein, Eva Schildmann, SedPall Study Group

**Affiliations:** 1 Department of Palliative Medicine, LMU University Hospital, LMU Munich, Munich, Germany; 2 Palliative Medicine, Faculty of Medicine, University of Augsburg, Augsburg, Germany; 3 Comprehensive Cancer Center Alliance WERA (CCC WERA), Würzburg, Erlangen, Regensburg, Augsburg, Germany; 4 Institute for History and Ethics of Medicine, Interdisciplinary Center for Health Sciences, MLU Halle-Wittenberg, Halle-Wittenberg, Germany; 5 Institute for Ethics, History and Theory of Medicine, Medical Faculty, University Münster, Münster, Germany; 6 Department of Palliative Medicine, University Hospital Erlangen, Friedrich- Alexander-Universität Erlangen-Nürnberg (FAU), Erlangen, Germany

**Keywords:** Deep sedation, Hypnotics and sedatives, Palliative care, Qualitative research, Terminally ill, Decision making, Informed consent, Patient participation

## Abstract

**Background:**

The decision-making process for sedation in palliative care remains under-researched, with evidence of limited involvement of patients and their relatives despite guidelines. The aim of this study was to explore the decision-making process for sedation in specialist palliative care in Germany, including all types of sedation (light to deep, temporary or continuous (until death)).

**Methods:**

Qualitative semi-structured interviews with 26 physicians, 22 nurses, eleven other members of the multiprofessional care team, eight relatives, and six patients. Recruitment took place via contact person in ten palliative care units and seven specialist palliative home care services in Germany. We analysed the transcripts by Framework Analysis and applied the shared treatment decision-making model by Charles et al.

**Results:**

Findings could be assigned to the adapted 5-phase decision-making process: (1) In the initiation phase, preemptive discussions were typically limited to patients with chronic diseases or potential catastrophic events, with some physicians avoiding early discussions due to fears of pressure. (2) During information exchange, the amount of detail varied by sedation type, with often only little information given for mild forms. (3) In the deliberation phase, informed consent was more common for deep sedation, and some team members criticized inadequate documentation of consent. (4) Decisions to start sedation were usually collaborative, though challenges arose when there was no defined starting point for deep sedation. (5) Re-evaluation was partly described to be challenging due to concerns about reintroducing suffering if sedation was reduced.

**Conclusions:**

This study highlights the processual nature of the decision-making process for sedation in palliative care and proposes re-evaluation as a fifth phase. It underscores the importance of early communication, addressing professionals’ concern, and supporting shared decision-making throughout all phases.

**Supplementary Information:**

The online version contains supplementary material available at 10.1186/s12904-026-02029-9.

## Background

Sedation in palliative care is a sensitive topic due to its ethically complex nature [1]. The European Association of Palliative Care (EAPC) defines *palliative sedation* in their revised recommendations as “monitored proportional use of medications intended to reduce consciousness in patients with life-limiting disease” to relieve refractory suffering [2]. The reduction of consciousness can vary from light to deep and can be temporary or continuous (until death). Reported prevalence rates vary considerably, e.g. depending on the methodological approach and the care setting [3]. In Germany, there are only scarce recent data. One recent congress report showed that 5.3% of patients in German specialist palliative care received *palliative sedation *[4], and another study that up to 50% of hospitalized patients in Germany received sedative medications with continuous effect at the end of life – however, without a determination of the sedative effect of the medication being possible [3]. For an ethically sound practice, an adequate decision-making process is important. Decision-making in this context refers to the process of reaching an agreement about the initiation, implementation, and continuation of sedation between patients or, where appropriate, relatives and legal representatives and healthcare professionals. This includes the information, involvement, and consent of patients and potentially their relatives/legal representatives [2, 5–8]. Yet, available evidence suggests respective shortcomings: one study reported that 72% of patients did not provide informed consent to *palliative sedation *[9] and another that patient consent is often neither sought nor obtained [8]. The involvement of relatives in decision-making processes may occur more frequently than of patients [9], especially when their consent is considered a prerequisite for patients lacking cognitive capacity [6], but is also reported to be irregular [10]. A recent review showed that involvement of patients in the decision-making process is limited because it is performed “very or too late in the disease trajectory”. [6] These results are not only in contrast to guidelines on sedation in palliative care [2, 5, 11], but also to patient´s preference to be informed and involved in decision-making [12, 13]. It is notable that reports on the decision-making process are scarce and rarely the focus of currently published articles [6]. Robijn et al. presented findings on the decision-making process from the UNBIASED-Study conducted in Belgium, the Netherlands, and the United Kingdom [8]. Based on Charles et al.’s model of shared treatment decision-making [14], they identified four stages in the decision-making process: (1) initiation, (2) information exchange, (3) deliberation (4) decision to star [8]. The degree of patient involvement was found to vary, and early and regular discussions about patients’ preferences were identified as a point for improvement [8]. Another recent qualitative study in five countries described the decision-making as complex and iterative process with the decision to start palliative sedation being emotionally difficult for all parties [7]. The UNBIASED-study is limited to continuous deep sedation until death, which represents only one form of sedation in palliative care, and both studies are limited to cancer patients [7, 8]. Furthermore, existing studies indicate that sedation practice is highly influenced by cultural factors [7–9]. Cultural factors include for example communication norms (e.g. regarding truth-telling), understanding of autonomy, and the roles of patients and their relatives in decision-making [7, 9].

To address the described existing gaps in the literature, we aim to explore the decision-making process for sedation in specialist palliative care in Germany, including all types of sedation (light/deep, temporary/continuous) and diagnoses. As called for by previous studies our focus will be on how involvement of patients and their relatives in the decision-making process is understood and enacted [6, 8], drawing on the perspectives of healthcare professionals and complemented by accounts from patients and relatives.

## Methods

### Design

This study is part of the larger mixed-methods multicenter study ‘SedPall’, aiming to understand sedation practices in specialist palliative care in Germany and develop best practice recommendations [5]. We present findings from the qualitative part of the study, which includes accounts from healthcare professionals, relatives, and patients. We used the stages identified by Robijn and colleagues [8] for continuous deep sedation until death as framework for data analysis and interpretation, which are grounded in Charles et al.’s. model of shared treatment decision-making [14]. We applied the model as it provides a structured, empirically grounded framework for understanding decision-making in the context of sedation in palliative care. Using this approach enabled us to explore the applicability of the phases to the German socio-cultural context and all types of sedation, which supports comparability and theory development in an area where few established conceptual frameworks exist. The Framework Analysis method was applied to examine a range of perspectives, while enabling a detailed examination and comparison within categories [15]. We employed a constructivist epistemological stance and an idealist ontology in accordance with the aim of the study and due to the paucity of qualitative data on sedation practice in Germany.

### Setting

All participants were recruited from ten palliative care units (hospital wards providing specialist palliative care) and seven specialist palliative home care teams, located in twelve cities in different federal states of Germany. Specialist palliative care refers to care provided by multiprofessional teams with advanced expertise, addressing complex symptom burdens and psychosocial needs of patients with life-limiting diseases, such as palliative care units and specialist palliative home care teams [16]. The involvement of both hospital and home care settings in different cities in Germany was necessary to inform the development of national best practice recommendations, which was the overall aim of the consortium project.

### Population

We interviewed three distinct groups:

1. Healthcare professionals (HCPs) in specialist palliative care (SPC): nurses, physicians, and other members of the multiprofessional team (physiotherapists, social workers, psychologists, spiritual carers). Participants were included if they had at least experienced one case of sedation in the last year in SPC.
2. Relatives of patients who had received or were currently receiving sedation. Relatives of deceased patients were also included if the death of the patient was recent (< 12 months). By the term “relative” we mean all persons close to the patient regardless of their degree of kinship, including legal representatives.
3. Patients who had either been sedated, were receiving sedating medication (resulting in light and/or temporary sedation), or had been informed about / made plans for the possibility of deep continuous sedation as possible future treatment option based on their disease trajectory.

### Sampling

We used a maximum variability sampling strategy for HCPs, aiming to include a wide variety of perspectives of professionals involved in the process of sedation in palliative care. This sampling approach involves deliberately selecting participants who differ widely in relevant characteristics [17]. Characteristics for sampling included the setting, the location of the setting, profession and position, and, as far as possible, years of experience, age and gender, leading to a predetermined sample size of 50 to 60 participants. For relatives and patients, we chose convenience sampling, as we were dependent on the participating centers for their recruitment. Here participants were selected based on their ease of access and availability, rather than through systematic criteria.

### Recruitment

Recruitment took place in the participating palliative care units and specialist palliative home care teams of the SedPall study. In each center a contact person was appointed, who informed potential participants (HCPs, relatives, and patients) about the study and asked if they would agree to be contacted by a member of the research team. Interested participants were contacted via email or telephone to schedule an interview appointment.

### Data collection

We conducted semi-structured interviews. Different interview guides were developed for each group (HCPs, relatives, patients) and subgroups (nurses, physicians, other HCPs), based on the literature and clinical as well as legal and ethical experience of the SedPall-consortium. The interview guides were discussed with experienced qualitative researchers in the department and a qualitative expert group at LMU University as well as with members of the Patient and Public Involvement (PPI) group. They covered the whole process of sedation, including one main section with questions pertaining to the decision-making process leading to sedation. All participants were asked detailed, open-ended questions on this topic. The complete interview guide for healthcare professionals is provided as supplementary material (Supplementary Table 1). Two trained researchers (VH, JB) conducted the interviews between July 2018 and September 2019. The interviews with HCPs and relatives lasted approximately 60 min, while those with patients lasted 20 min. Interviews were audio-recorded and transcribed verbatim, including anonymization. Using a questionnaire, we collected sociodemographic information of the participants. Data saturation was monitored throughout the analysis process and no new themes emerged after the planned number of interviews had been conducted.

### Data analysis

We used the Framework Approach to analyze the interviews using the qualitative analysis software MAXQDA version 2018.2. Framework Analysis facilitates the analysis of a high amount of data and the comparison of data within categories as well as within cases, thereby enabling the acquisition of a more comprehensive understanding of a broader range of perspectives on sedation [15]. Two researchers (VH and JB) independently analyzed a subset of the interviews and organized their indices in a first framework. Together, they developed an initial analytical framework for each participant group with support from ES. Categories were derived both inductively and deductively, based on the themes from the interview guides. These frameworks were refined while indexing the remaining interviews (JB, SM, JG). The final framework consisted of twelve categories with up to ten sub-categories. One category, which will be focused on here, was “decision-making process”. We constructed our data based on the 4-stage model developed by Robijn and colleagues [8] by organizing them in accordance with the chronological stages, thereby adapting the model to our data. To assure rigor, project progress and results were continuously discussed within the SedPall Study Group, consisting of researchers with multidisciplinary backgrounds, including clinicians, and members of the public in our PPI group. We followed the Consolidation Criteria for Reporting Qualitative Studies (COREQ)-guidelines [18].

## Results

We conducted interviews with 26 physicians, 22 nurses, eleven other members of the multiprofessional care team, as well as eight relatives and six patients (see Tables 1, 2 and 3). In addition to the four stages of decision-making identified by Robijn et al. [8] for continuous deep sedation until death, we identified a fifth stage, the re-evaluation phase. The five-stage structure was developed inductively, based on participants’ accounts. We could assign all our findings, which relate to the whole range of sedation in palliative care, to this adapted 5-stage-model: (1) initiation phase where the issue is raised, (2) exchange of all necessary information, (3) deliberation phase in which it is decided to use sedation when it becomes appropriate (4) decision to begin sedation and (5) re-evaluation.

**Table 1:** Details on the interviewed healthcare professionals

	Physicians *n* = 26	Nurses *n* = 22	Other professions *n* = 11	Total *n* = 59
Gender				
Female, n (%)	15 (58)	18 (82)	8 (73)	41 (69)
Work experience in years, median (range)	17 (4–36)	24 (2–40)	23 (1–20)	23 (1–40)
Completed structured training in palliative care, n (%)				
Yes	20 (77)	20 (91)	9 (82)	49 (83)
Intensive care unit / intermediate care experience, n (%)				
Yes	22 (85)	6 (27)	n/a	28 (48)
SPC^a^ Setting, n (%)				
Palliative care unit	11 (42)	12 (55)	7 (64)	30 (51)
Homecare	9 (35)	8 (36)	1 (9)	18 (31)
other/NR^b^	6 (23)	2 (9)	3 (27)	11 (18)
Medical specialty, n (%)				
Internal medicine	9 (35)			
Anesthesiology	7 (27)			
General Practice	4 (15)			
Neurology	3 (11)			
Others	3 (12)			

^a^Specialist palliative care

^b^NR: Not reported; other: For example, spiritual carers who worked for the whole hospital rather than just one ward

**Table 2:** Details on the interviewed patients

	*n* = 6
Gender	
Female, n	3
Age in years, median (range)	69 (50–95)
SPC^a^ Setting, n	
Palliative care unit	2
Homecare	4
Primary Diagnosis group, n	
Malignant neoplasm	4
Circulatory system	1
Nervous system	1
Sedation, n	
Had Received	2
Planned	4
Types of sedation received^b^, n	
Continuous	1
Temporary	1
Deep	1
Light	1

^a^Specialist palliative care

^b^Numbers do not add up to the total number of patients who had received sedation as they may have received more than one type of sedation

**Table 3:** Details on the interviewed relatives

	*n* = 8
Gender	
Female, n	6
Age in years, median (range)	47 (35–57)
SPC^a^ Setting, n	
Palliative care unit	5
Homecare	3
Degree of kinship, n	
Partner	2
Children	2
Friends	2
Other	2
Types of sedation^b^, n	
Continuous	8
Temporary	0
Deep	5
Light	4
Patient died under sedation, n	
Yes	6

^a^Specialist palliative care

^b^Numbers do not add up to the total number of relatives as patients may have received more than one type of sedation

### Initiating the conversation about sedation

The accounts of the participants indicated that conversations about sedation were initiated by any of the involved groups (patients, relatives, HCPs). Patients with non-malignant diseases, such as motor neuron disease, or with high risk for sudden catastrophic events (e.g. terminal bleeding) were commonly informed preemptively of the possibility of deep (continuous) sedation by the HCPs and asked for their “advance” consent should the need arise. This prior information and consent of the patients often took place when they initiated a discussion of their options in the context of a ‘worst-case-scenario’ related to their diseases. For other patient groups and other types of sedation, no clear criteria for initiating preemptive discussions could be identified. Fears that patients might pressure physicians to administer sedation in cases without adequate indication were reported by HCPs as reasons for not discussing the option in advance. Others saw an early presentation of the option as reassurance for the patients.

*Brigitte (physician): “And then the first conversation was about palliative sedation. Interestingly*,* he also landed on the palliative care unit a few months later but did not have a sedation. This means*,* until the end*,* only the knowledge that it is a possibility was enough for him […]. But I also have experienced other patients*,* where we also informed them early about it*,* that it was a possibility*,* and who*,* in the same conversation*,* demanded: “yes*,* right this instantly”. […] So*,* to get pressured*,* the moment you mention it*,* that is a difficulty*,* that is not something you want. On the other side*,* for some patients it can provide safety.”*

Patients´ accounts also indicated that discussing sedation in advance as a means of providing reassurance was perceived as important.

*Darius (patient): “[…] I also know*,* how people with lung cancer*,* how painful the dying of these people is. I’ve heard of several cases also from my circle of family and friends*,* and they’re terrible. And I’m glad I’m so well safeguarded now.”*

### Information exchange

**Fig. 1:**
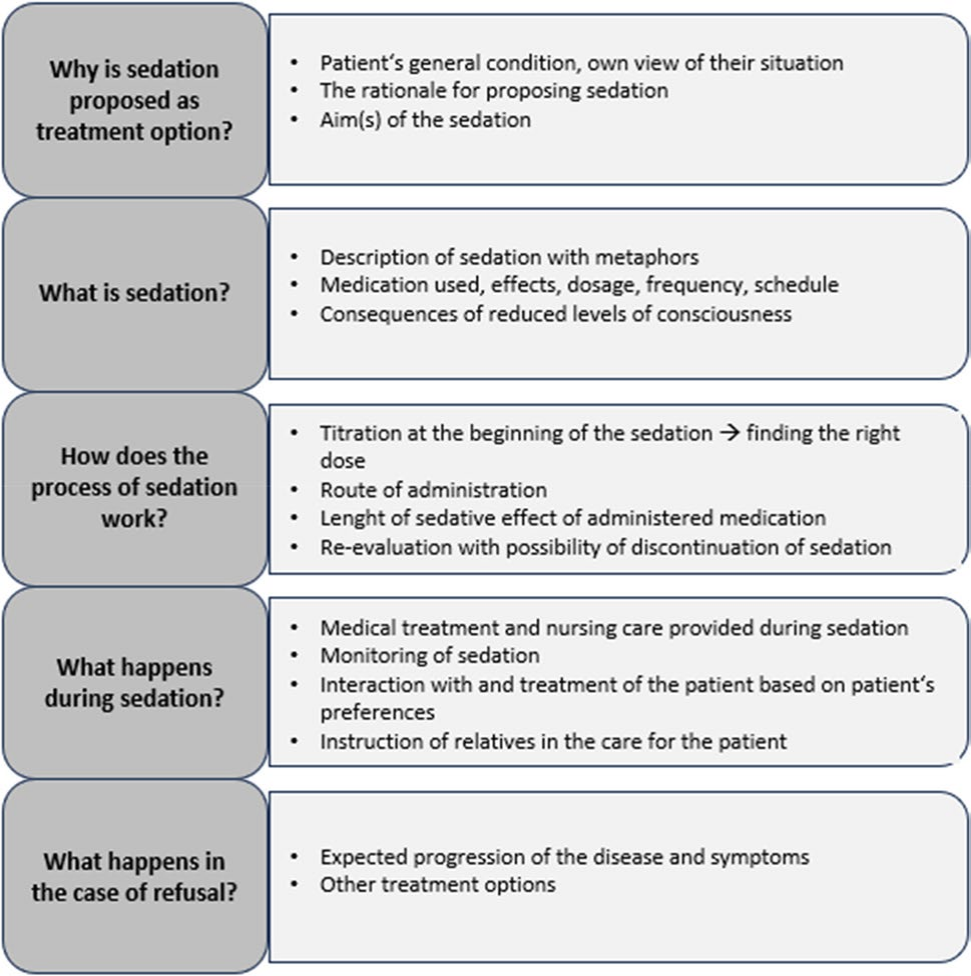
Contents of information exchange with patients and relatives pertaining to sedation, according to the interviewees´ accounts

Most HCPs described that they provided information pertaining to aims, risks and benefits, the process, and practical issues in relation with sedation (see Fig. 1). For example, when informing patients about why sedation is proposed as a treatment option, HCPs most often mentioned symptom control as aim. The consequences described were often related to the reduced ability to communicate or reduced oral intake.

The amount and nature of the information provided to the patient depended on the individual case and on the type of sedation proposed: milder forms of sedation often prompted almost no discussion or information exchange, while deeper forms of sedation were talked about more thoroughly. For example, information about what happens during sedation was mostly discussed only in cases of continuous deep sedation, as were decisions related to it such as hydration or nutrition.

Some participants described this lack of information sharing for milder forms as potentially problematic, as patients and/or relatives may be confused about the reduction of consciousness, such as it was described by Frida (relative) and Alice (HCP):

*Frida (relative): “Well*,* I think during the first stay*,* […]*,* it wasn’t quite clear whether it was due to the disease or the medication that he was so sleepy. We probably would have needed more know-how. But I don’t want to say that it wasn’t addressed. There is so much (laughs) that you are confronted with*,* but I think more clear information would actually be useful.”*

*Alice (other profession): “These short-term sedations are not always discussed in detail with the patient. Instead*,* they say: Yes*,* he can have an on-demand medication. And the patient then says: Yes*,* yes*,* I feel so bad*,* I really want that medication. But that’s the kind of drug that really knocks him out. It also happens that patients tell me afterwards: “I don’t know*,* I’m completely out of it. I am so tired*,* I am/why am/why am I so tired? Why am I so weak? Why am I asleep all the time?” So that sometimes it is met with a bit of a lack of understanding. So what the effect of such a drug really is. We/I have experienced that quite often.”*

When describing the information exchange, it is also important to evaluate how HCPs and patients describe and therefore understand sedation. Participants described sedation as a state of sleep, anesthesia, or coma. When talking to patients or relatives, the sleep-metaphor was used most often by HCPs. Some physicians explained that they use this image because sleep is usually positively connotated in the minds of relatives. Explanations such as “*He is resting*,* he is fine. […] And then they would simply sleep through their own death.”* (Gemma, physician)*”* echo this intention.

### Deliberation and the decision to use sedation

The timeframe between initiating the decision-making process and reaching a shared decision was determined by the urgency of the situation: the burden of symptoms and the perceived suffering of the patient. HCPs reported instances of immediate decision as well as waiting times of several days to weeks.

The practical aspects of informed consent differed between institutions and individual HCPs as well as depending on the type of sedation: Obtaining informed consent was described more for deep types of sedation. Depending on the institution, consent could be given only verbally or in written form or had to be documented by the HCP and/or witnessed by a second HCP. Some HCPs criticized that consent is often not adequately documented in patient records.

The HCPs raised the following problematic issues for the informed consent of the patient:

i) The agreed upon (and consented) sedation process might have to be adapted and changed over time

Consent was described problematic, when sedation is not deliberate and planned, but happens in a gradual process of increasing the dose of sedative drugs in response to the increasing suffering. In such situations, decision-makers might repeatedly agree to each individual administration of sedatives, but an increased dose and/or frequency of administration of sedatives may eventually lead to a state of continuous deep sedation the patient has not consented to.

*Stefanie (physician): “Yes*,* exactly. As a rule*,* when you see signs of restlessness and anxiety*,* you start to apply anti-anxiety medication on a regular basis. And if that’s not enough*,* then the single doses are increased. And at some point*,* however*,* it is the case that a single dose is quite high and the patient´s condition worsens after it has subsided and becomes agitated again*,* so that one then simply sees the need to apply it continuously in order to simply dose it evenly. By then*,* however*,* the patient is often already in such a state that it is no longer possible to discuss the matter with him or her due to medication or illness. Then it becomes a question of the presumed will of the patient. These are things that have hopefully been discussed in advance.”*

ii) The influence of the symptom burden on the patient´s ability to make informed choices

Some participants put into question a patient´s ability to make informed decisions in a situation of extreme distress:

*Emilia (physician): So*,* if someone has severe symptoms*,* is he free to make decisions at all?*

iii) Patients` refusal of sedation

HCPs experienced the refusal of sedation often as very difficult, especially when they must witness great suffering.

*Paul (physician): “[…] I think it also depends on how much I can bear myself. Yes*,* if I now see that someone is extremely agitated*,* is in extreme agony*,* the question is*,* does patient autonomy then always take precedence over everything or may I then possibly use a sedative medication*,* even if I have explicitly received no prior authorization from the patient? […] But I really have to accept that.”*

*Quartilla (nurse): “Sometimes you also have such a high level of suffering as a team that you think the patient´s symptoms are poorly controlled […] so rather the other direction*,* where our level of suffering is so high*,* and we think we can do this better […] then you have to endure this as a team […]. That somebody just endures this pain and would rather not take medication.”*

### Decision to start sedation

The start of deep types of sedation was usually decided by the patient and HCPs together. If the patient was no longer competent, HCPs made the decision together with the relatives. An emergency could also mark the starting point, e.g. in case of crisis sedation. Patients´ wishes played an important role. Patients may for example wish to organize their affairs, wait for a relative or take time to say goodbye.

*Zora (relative): “And she [young daugther] also agreed to it and just said*,* “But before I want to see grandma and grandpa and everyone who is here.” […] the first thing she said was*,* “I have to have my engagement ring and I want to say goodbye to everyone.” And that was the way it was*,* so we got grandma and grandpa and everyone was allowed to see [name of daugther]*,* so to speak*,* individually*,* have a conversation. And there was the first laugh again. When my mom left*,* [name of daugther] called after her*,* “Grandma*,* see you soon.” So*,* it’s not that there can’t be moments that are funny in this terrible situation.”*

HPCs described the challenge when there is no defined starting point for deep types of sedation, but an increased dose and/or frequency of sedatives administration may eventually lead to a state of continuous deep sedation (see also the quote from Stephanie in 1.i)).

### Re-evaluation-phase

Participants described re-evaluation as a distinct decision point after the start of sedation, involving renewed assessment and – where feasible – re-confirmation of consent. Once sedation has been administered, participants described two instances in which consent could be withdrawn or renewed:

i) During titration:

When sedation is started, the medication is titrated to the dose which alleviates the patient´s suffering to an acceptable/comfortable level. Outside of crisis sedation where the dose is rapidly increased to unconsciousness, HCPs describe titration as a process which is undertaken in close consultation with the patient and over a longer timeframe. The depth of sedation is tailored to the patient´s needs:

*Ute (physician): “And exactly*,* then also this titration*,* so to speak*,* that one says that the depth of sedation is not started abruptly*,* but slowly*,* yes. And that you can also reduce it again*,* as it were*,* yes*,* that simply all these things are clear for all those who are involved*,* yes.”*

This is echoed by a relative describing sedation for her mother-in-law:

*Elisabeth (relative): “It started with a small dose*,* first of all to see how she tolerates it*,* I can still remember the conversation: “That will change her a little bit and if you don’t want to take it*,* we can always stop it or dose it differently” […]. They said she didn´t have to take it and my mother-in-law really liked to take it and as she got worse*,* she also deliberately wanted to experience less.”*

ii) After the initial titration: re-establishing alertness

Re-evaluation of sedation after initial titration is either possible because the effect of the medication wears off (after a single or repeated administration) or sedation is intentionally reduced - after a pre-defined interval to a degree where patients can communicate again. According to the interviews re-evaluation served five purposes (see Table 4).

**Table 4:** Purposes for re-evaluation

• Check if suffering persists • Ask how the patient experienced sedation • Confirm that the patient wishes to (dis)continue the sedation • Adapt schedule of sedation according to the patient´s expressed wishes: define phases of increased/decreased alertness (e.g. according to day-night-rhythm or planned visits from family or friends) • Re-evaluate depth of sedation	

Problems arise when a re-evaluation is scheduled but not performed because of the fear that reduction of sedation might re-introduce intolerable suffering. In such cases, relatives are usually included in the decision to forgo re-establishing alertness in favor of continuing sedation. This was often experienced as a burden:

*Frida (relative): “And we also had in the evening*,* when the sedation started at five o’clock*,* there was also: “Yes*,* then we’ll just see how it looks in the morning.” Because*,* so far*,* it was always switched off again early and that was now for me personally just very difficult*,* because then the doctor and I decided […] that we keep it running. So then because she also said to me*,* “That would really be absolutely not good for him now if we take him out of there.”*

### Summary of results

The most important results from our study and Robijn’s study on the respective phases are summarised in Table 5.

**Table 5:** Comparison of the key results of the study from Robijn et al. and the SedPall study

Decision-making phase	Key findings from Robijn et al. (Continuous sedation until death; cancer patients)	Key findings from SedPall (All types of sedation and diagnoses)
1. Initiation	Conversation initiated by physician or patient, varies by country. In the Netherlands and Belgium, some patients initiated conversation by requesting euthanasia.	Initiation varied: patients, relatives, and HCPs^a^ reported initiating discussions. HCPs^a^ often avoided early discussion due to fear of pressure, except for patients with non malignant diseases or in high-risk cases. Early discussion of the option of sedation can provide reassurance for patients and relatives.
2. Information exchange	Physicians in the Netherlands and Belgium predominantly provided information on the possibilities and indications for sedation, leaving the decision to the patients. In the UK, physicians took the lead by proposing the possible use of sedation, in the hope of providing the necessary information and eventually obtaining patients’ consent.	Depth and timing of information varied by the type of sedation: light sedation was often not explained sufficiently from the patients’ and relatives’ perspective. HCPs^a^ most often mentioned symptom control as aim. Metaphors like “sleep” were commonly used.
3. Deliberation	Often a rather difficult decision for patients and relatives. In the Netherlands and Belgium, the discussion sometimes focused on the difference between palliative sedation and euthanasia. In the event of disagreements between patients and their relatives, physicians emphasised the patients’ wishes.	Informed consent was more often sought for deep sedation. The timeframe for makign the decision depended on how urgent the situation was. Concerns were raised about the patient’s cognitive capacity to give informed consent, patients refusing sedation, and the quality of the documentation.
4. Decision to start sedation	The decision to start continuous sedation depended on whether the patient had decision-making capacity. In Belgium and the Netherlands, competent patients often initiated the decision to start sedation themselves, or decisions were made in advance with patient or family input. In the UK, sedation was usually introduced gradually by clinicians without a clearly defined decision point.	Start often collaborative; patients’ wishes (e.g. timing, chance to say goodbye) taken into account; challenges when no clear starting point.
5. Re-evaluation *(new phase)*	– *(not included in Robijn’s model)*	Re-evaluation allows patient re-involvement. Problems when re-evaluations are scheduled, but not performed due to fear of re-appearance of intolerable suffering; taking part in these decisions experienced as burden by relatives).

^a^Healthcare professionals

## Discussion

In line with literature on decision-making for sedation in palliative care, we found the process to be challenging and complex [6–8]. Breaking it down into different phases according to Robijn et al. [8] helped to clarify the process and facilitates comparison between studies, and therefore between countries. We added a new fifth phase describing the process after sedation was initiated: the re-evaluation-phase, where patients can confirm or withdraw consent or modify sedation conditions. Assigning our findings to these five phases clarified the challenges of decision-making.

### Applicability of the decision-making model and socio-cultural comparison

Using existing scientific theories to research medical decision-making helps explain underlying mechanisms and improves the validity and generalizability of the results [19]. Robjin and colleagues [8] developed their four-phase model based on the shared treatment decision-making model by Charles et al [14]. Although Charles’ model dates from 1999, it remains useful for structuring the decision-making process for sedation. Robjin and colleagues added the “initiation phase” to understand who initiates the possibility of sedation [8], and we added another phase, the re-evaluation phase. In this phase, patients can make informed choices based on their experiences. Participants gave multiple reasons for titrating, gradually reducing, and discontinuing sedation. The re-evaluation phase may be particularly relevant in cases where symptom burden is dynamic and/or potentially reversible (e.g. in cases of psychological or existential suffering) when the disease trajectory is uncertain, or when doubts arise among HCPs, patients and/or relatives regarding the indication for sedation. It offers a structured opportunity to reassess the situation, especially in light of ethical concerns or diverging views [2, 5]. While re-evaluation is widely recommended in guidelines and clinical practice frameworks [2, 5], to our knowledge no quantitative data are available on how often sedation is discontinued and patients regain the ability to make informed choices in clinical practice. Moreover, it should be noted that re-evaluation is not always feasible, particularly when patients are in the dying phase and naturally becoming less responsive or unconscious due to disease progression. The approach of titrating and re-evaluating is similar to that reported by HCPs from the UK, as opposed to rapidly increasing the dose, which was more often reported by HCPs from Belgium and the Netherlands [8]. It is important to notice that involvement of patients and their relatives is not limited to the general decision to use sedation, but also to determining the starting point of sedation, echoing existing findings [7, 8]. Additionally, we found that involvement can take place at further points in the decision-making process (see Fig. 1), e.g. the preferred treatment under sedation. This involvement reinforces a sense of control and autonomy for patients and their relatives, also highlighted by the EAPC guideline [2]. Finally, the use of the sleep metaphor to compare sedation to a state of sleep was widely used by our participants. Dying in one´s sleep is viewed by many as a good death [20]. Comparing sedation to a positively connotated, natural state like sleep rather than anesthesia may influence patients towards sedation 21 . While commonly used in medicine [22], the effects of metaphors are rarely studied. Robijn et al. also found this metaphor’s use [8], suggesting it is not context specific.

### Challenges and areas for improvement

HCPs found it challenging when patients refused sedation despite severe suffering, creating conflict between respecting autonomy and other obligations towards the patient. The EAPC-guideline addresses staff distress from administering sedation but not from patient refusal [2]. Measures to alleviate such distress should be developed [23, 24]. Contrary to patients’ preferences, involvement of patients and their relatives in decision-making is often limited or impossible because it is initiated too late [6]. While a recent qualitative study contradicted this evidence [7], it was confirmed in our study, except for patients who were expected to experience sudden catastrophic events. HCPs stated a possible reason for their hesitancy to suggest sedation to patients: the fear of patients or relatives demanding this option without a valid indication and thus pressuring physicians. In this context, it is important to consider whether such concerns might be influenced by societal debates or legal developments surrounding voluntary assisted dying. At the time of data collection (2018–2019), assisted suicide was restricted under German law (§ 217). The ending of patients’ life was at that time of the study (as it is now) legally prohibited. Based on our analysis, there does not appear to be a direct link, and available evidence does not allow for a direct comparison of patients’ or relatives’ request rates in relation to the legality of assisted dying [25–27]. This could become a relevant aspect in future research, particularly given recent legal changes in Germany. When involvement is delayed, adequate progression through the necessary stages is prevented. Particularly, this is the case when intolerable suffering at the end of life may affect patient’s decision-making capacity, as described by HCPs. Recent studies documented this reduced capacity [28, 29], which HCPs struggle to assess accurately [28, 30]. In situations where patients experience severe suffering and reduced decision-making capacity, HCPs may need to weigh patient autonomy against the principle of acting in the patient’s best interests. Ethical frameworks emphasize that in such cases, decisions should aim to alleviate suffering while respecting the presumed values and preferences of the patient [31, 32]. Developing coping strategies and guidelines for HCPs, similar to those by the German Society for Palliative Medicine for managing patients’ wishes to die, could empower HCPs to initiate earlier discussions about end-of-life options, facilitating a more adequate decision-making process [33]. While best practice involves adapting to patients’ informational needs, not all patients and, where appropriate, relatives in our study were sufficiently informed. For example, patients or relatives of patients receiving lighter forms of sedation reported being surprised by side-effects. Gradual transitioning to continuous deep sedation without a clear starting point posed additional challenges for an adequate information exchange by HCPs, as initial information provided typically pertained to the administration of single doses of sedatives rather than the continuous administration. Additionally, HCPs emphasized the need for better documentation of consent in patient records. Documentation standards for eliciting informed consent may be helpful in this regard [34], preventing different approaches between settings [7]. A checklist for information to be provided, amongst other tools supporting best practice of sedation, has been developed in the iSedPall project [35]. Finally, the EAPC-guideline emphasizes that HCPs must inform both the patient and their relatives of the potential instability of the situation when reevaluating alertness, including the risks of symptoms returning or death [2]. Relatives experienced it as distressing when alertness could not be re-established and they were asked for their assent. Therefore, it is crucial to also discuss the patient’s preferences for this situation in advance. We included agreements regarding potential awakening attempts or the decision to forgo such attempts in the SedPall-recommendations [5].

### Strengths and limitations

The validity of this study is increased by data collection across 17 specialist palliative care institutions, including diverse professional perspectives, relatives and, uniquely, SPC patients with first-hand experience of sedation. Although we aimed for diversity by including institutions from different regions (urban and rural) and perspectives (clinical, ethical, legal), the full cultural diversity of Germany is not represented. In particular, patients and relatives without sufficient German language skills were not included. The number of interviewed patients and relatives was comparatively small, resulting from the broader SedPall study requirement to include a wide range of settings and professional backgrounds as well as challenges to recruit eligible patients and relatives. Patients’ often critical health status presented a challenge, yielding short interviews. As most accounts stem from HCPs, descriptions of patients´ and their relatives´ involvement and of informed consent may be subject to perspective and social desirability bias. Moreover, a potential selection bias for HCPs cannot be ruled out as participants with more experience or stronger views on sedation in palliative care may have been more inclined to participate. Our study focused on the perspectives of specialist palliative care teams and did not explore how other HCPs, such as general practitioners or nurses, are involved in the decision-making process. Future research should explore how collaboration between specialist and non-specialist providers can support timely and ethically sound decisions on sedation in these settings. The broad approach, covering all types of sedation practices, acknowledges sedation as a gradual process. We avoided imposing a predefined definition, instead using participants’ own understanding of sedation. However, this also meant that it could not always be distinguished which type of sedation they were referring to in their accounts. Although data collection took place prior to the 2023 revision of the EAPC framework, we consider the findings to remain relevant. Attitudes and decision-making processes in palliative care tend to evolve slowly. Furthermore, our findings align well with the emphasis of the revised EAPC framework. The insights gained from this study contributed to the recent development of national guidance in Germany.

## Conclusion

This study contributes to a more nuanced understanding of the decision-making process for sedation in palliative care by highlighting its processual nature and proposing a fifth phase, the re-evaluation. This phase reflects clinical practice where sedation may be titrated, paused, or discontinued, allowing for ongoing assessment and, in some cases, renewed patient involvement. Our findings show that improving patient and relative involvement requires addressing HCPs concerns (e.g., fear of inappropriate sedation requests), enhancing communication about the possibility and process of sedation, and supporting shared decision-making throughout all phases. To support patients’ autonomy and reduce uncertainty in acute situations, the option of sedation can be addressed as part of Advance Care Planning conversations. This may enable patients and their relatives to reflect on preferences regarding indications for sedation, the possibility of re-evaluation, and the desired depth and duration of sedation before critical situations arise. These insights provided a practical foundation for developing best practice recommendations in the SedPall project, aiming to guide HPCs in structuring decision-making processes and fostering patient-centred care in this ethically sensitive context.

## Supplementary Information

Supplementary Material 1.

## Data Availability

The datasets generated of analysed during this study (transcripts as well as central and thematic charts) are available from the corresponding author on reasonable request, under the provision that the research ethics committee of the Medical Faculty at Ludwig-Maximilians-University Munich agrees.
