# Supplementary material for: The decision-making process for sedation in specialist palliative care: a qualitative interview study with team members, relatives, and patients
Source: BMC Palliat Care. 2026 Feb 24;25:75. doi: 10.1186/s12904-026-02029-9 (PMC13037086; doi:10.1186/s12904-026-02029-9)
Supplement: Supplementary file 1 — Supplementary Material 1. [file 12904_2026_2029_MOESM1_ESM.pdf]

**SedPall – Interview guide for healthcare professionals: physicians***

| Key questions (narrative prompt) | Check - Memo for possible follow-up questions |
|---|---|
| **Part 1: Experiences with sedation in specialist palliative care and understanding of the concept** | |
| What experience have you gained with sedation in your work environment? | Experiences<br>Descriptions<br>Emotions, own experiences<br>Protocol/Guidelines |
| Can you describe in your own words what distinguishes sedation in palliative care for you? | Definition/Understanding<br>Different types of sedation/spectrum of sedation measures<br>Terms such as palliative sedation, terminal sedation, continuous/intermittent |
| **Part 2: Indication** | |
| When do you usually sedate a patient? | Conditions<br>Time in the course of the disease<br>Intention/aim<br>Restraint<br>Patient's request<br>Refractory symptom<br>(Unbearable) suffering<br>▪ Subjective vs. objective suffering<br>▪ Difference between physical and psycho-existential symptoms as the cause of suffering<br>Demarcation: unjustified sedation |
| **Part 3: Decision-making and consent** | |
| Based on your experience, how is the decision made to sedate a patient? | Allocation of roles in decision-making<br>Disagreement/conflict situations<br>Time pressure during decision-making due to a high burden of symptoms?<br>Weighing the benefits/risks<br>Weighting awareness<br>Life-shortening effect |
| If you have informed the patient and, if applicable, their relatives in the past that sedation is being considered, how did you do this? | Informed consent of patient and, if applicable, relatives<br>Health care proxy<br>Judicial guardian<br>Contents of the informed consent discussion<br>Autonomy in the case of unbearable suffering |

| | |
|---|---|
| **Part 4: Challenges and opportunities**<br><br>What have you found particularly difficult or challenging in your experience of sedation?<br><br>What have you found to be particularly positive about sedation cases? | Nutrition and hydration<br>Checking the outcome<br>Letting the patient wake up<br>Monitoring<br>Effect on treatment team<br>Demarcation from euthanasia |
| **Part 5: Contextual aspects**<br><br>In your experience, do patients with and during sedation die differently from other patients in your daily practice? | Demarcation from “natural death”<br>Procedures/Rituals/Farewells<br>Perception of the patient |
| **Part 6: Conclusion/Reflection**<br><br>When you look back on our conversation: What was a particularly interesting or important aspect for you? Why?<br><br>Is there anything else you would like to say about this subject that we have not addressed? | Reflection on conversation |

*Interview guides were developed for nurses, physicians, and other members of the multiprofessional care team, with slight adaptions, respectively.
